# Quality and Reliability of Transarterial Chemoembolization Videos on TikTok and Bilibili: Cross-Sectional Content Analysis Study

**DOI:** 10.2196/73855

**Published:** 2025-09-17

**Authors:** Yushuo Niu, Guilan Song, Zheyu Niu, Sijian Xiao, Cuicui Li, Na Han, Hao Wan, Xiaohong Hou

**Affiliations:** 1 Department of Hepatobiliary Surgery, Shandong Provincial Hospital Affiliated to Shandong First Medical University, 9677 Jingshi Road, Lixia District, Jinan, 250000, China, +86-13290333867; 2 Qilu Hospital of Shandong University, Jinan, China; 3 The Second Affiliated Hospital of Shandong First Medical University, Taian, China

**Keywords:** transarterial chemoembolization, TACE, hepatocellular carcinoma, health education, short videos, quality analysis

## Abstract

**Background:**

Transarterial chemoembolization (TACE) is a widely used treatment for advanced, unresectable hepatocellular carcinoma, often requiring multiple sessions for optimal efficacy. TikTok and Bilibili have gained widespread popularity as easily accessible sources of health information.

**Objective:**

This study aims to assess the quality of the information in Chinese short videos on TACE shared on TikTok and Bilibili.

**Methods:**

In November 2024, the top 100 TACE-related Chinese-language short videos on TikTok and Bilibili (a total of 200 videos) were assessed and reviewed. Initially, basic information about the videos was recorded and analyzed. Subsequently, Global Quality Score and the DISCERN tool were used to evaluate the information quality and reliability of the videos on both platforms. Finally, multifactorial analysis was used to identify potential factors influencing the quality of the videos.

**Results:**

TikTok is more popular than Bilibili, despite its videos being shorter in length (*P*<.001). The quality of short videos on TACE found on both platforms was of low quality, with average Global Quality Score scores of 2.31 (SD 0.81) on TikTok and 2.48 (SD 0.80) on Bilibili, as well as DISCERN scores of 1.86 (SD 0.40) on TikTok and 2.00 (SD 0.44) on Bilibili. The number of saves (β=.184, *P*=.008; β=.176, *P=*.01) and days (β=.214, *P*=.002; β=.168, *P*=.01) since publication were identified as closely related variables to video quality and reliability. Furthermore, the duration of the video was closely related to its reliability (β=.213, *P*=.002).

**Conclusions:**

This study indicates that the quality of TACE-related health information in the top 100 short videos on both Bilibili and TikTok platforms is suboptimal. Patients should exercise caution when relying on health-related information from these platforms. Social media companies should establish review teams with basic medical knowledge. It is essential for the platforms to enhance their recommendation algorithms and implement measures for video quality assessment. Health care professionals should be aware of the limitations of these videos and work to improve their quality.

## Introduction

Hepatocellular carcinoma (HCC) ranks as the fourth most common cause of cancer-related deaths globally and poses a significant challenge to health care systems worldwide [1,2]. According to the Barcelona Clinic Liver Cancer staging system, transarterial chemoembolization (TACE) is the first-line treatment for intermediate and advanced HCC, including unresectable, multinodular HCC without extrahepatic spread [3]. TACE has been widely accepted and recommended across various clinical settings in Asian countries, demonstrating strong acceptability and favorable treatment outcomes [4]. However, the complexity of the procedure, potential adverse effects, and need for repeated interventions often leave patients with limited understanding and high emotional distress [5-8]. Therefore, enhancing public awareness and patient education about TACE is critical.

With the increasing prevalence of internet technology, the importance and ubiquity of social media use have risen, with an estimated global user base of 4.9 billion [8]. In China, TikTok and Bilibili are the top two video-based social media platforms, boasting hundreds of millions of users. According to the latest data from QuestMobile, TikTok’s monthly active users surpassed the 1 billion mark as of September 2024 [9]. This figure highlights the widespread acceptance and influence of short videos, emphasizing the public’s strong preference for fast and intuitive methods of information access. Short videos can be easily created and shared on these platforms, enabling users to interact through likes, comments, and bookmarks, which fosters broader participation across social groups, regardless of gender or age. Health care professionals and organizations have also leveraged these platforms to disseminate health-related information, promote healthy habits, and encourage disease prevention. These platforms present an unparalleled opportunity to improve public health literacy, enhance self-efficacy, and promote treatment adherence [10].

A recent systematic review of health information systems underscores accuracy and completeness as foundational data quality elements critical for informed decision-making [11]. While videos related to TACE are available on TikTok and Bilibili, providing accessible information to patients, the quality of these videos remains uncertain. Prior studies suggest that misinformation is common on social media and that users often lack the digital health literacy needed to evaluate content critically [12,13]. Therefore, understanding the sources of information, the characteristics of social media channels, and the content and framing of health information is crucial for navigating the dynamic landscape of TACE-related content on these platforms.

Content analysis achieves an objective and replicable examination of communication materials by encoding the content into predefined categories. This method enables quantitative and qualitative interpretation of media content, making it particularly suitable for evaluating health information on web-based platforms. Moreover, the Global Quality Scores (GQS) and DISCREN systems are widely used to assess the content, quality, and reliability of short videos related to various diseases on social media platforms [12,14-16]. Therefore, this study aims to investigate the top 100 videos related to TACE on TikTok and Bilibili, evaluating their content, quality, and reliability. Additionally, the correlation between video quality and factors such as video source, content, length, likes, comments, shares, and saves was analyzed and discussed. This analysis can help foster the development of social media platforms, offering the general public more comprehensive and appropriate ways to access TACE-related information. Ultimately, our goal is to promote the correct treatment of HCC.

## Methods

### Ethical Considerations

Shandong Provincial Hospital affiliated to Shandong First Medical University did not conduct ethical approval. The information in this study does not involve clinical data, human specimens, or experimental animals. All data were obtained from publicly available videos on TikTok and Bilibili. We complied with informed consent guidelines and have adhered to Chinese law and regulations regarding the protection of personal information, privacy, and human rights.

### Study Design

#### Search Strategy

**Figure 1.**
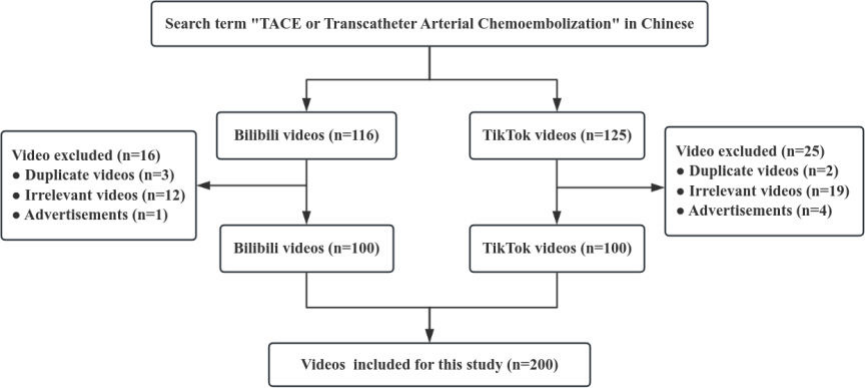
Search strategy for short videos on TACE. TACE: transarterial chemoembolization.

Videos were obtained from two platforms, Bilibili [17] and TikTok (Chinese version [18] on November 18, 2024. The search was conducted using the keyword “TACE” (Figure 1). The search was conducted through a mobile phone to ensure consistency and to avoid algorithm adjustments specific to desktop browsers. To minimize bias from personalized recommendation algorithms, we registered and logged into new accounts on each platform. Furthermore, we have disabled personalized recommendations in the platform settings.

The following filters were applied: (1) language restriction: Chinese only; (2) upload date: no restriction; (3) sorting method: “Comprehensive ranking,” which prioritizes videos with video completion rate, high engagement (likes, comments, and shares), and recent uploads. The exclusion criteria were as follows: (1) duplicate videos, (2) advertisement content, and (3) irrelevant content.

#### Sampling Procedure

We retrieved the top 100 videos based on overall rankings. This cutoff was chosen for three reasons: (1) it reflects common user experience, and focusing on the mainstream content recommended by the platform algorithm, and thus reflecting the current situation of health information dissemination in a more representative manner; (2) it balances depth and feasibility of manual coding across platforms; and (3) it is consistent with prior social media health content analyses, improving comparability [13,19,20]. There were no duplicate videos between the Bilibili and TikTok datasets.

#### Data Extraction

Two trained reviewers independently recorded. Data collected included the title, number of likes, number of comments, number of saves, number of shares, video duration, upload date, number of days since posting, uploader, video content, and video source. Disagreements were resolved by discussion or a third reviewer.

### Video Quality Assessments

Two widely adopted standardized scales were used. The GQS was used to assess the quality of information in the videos. The GQS consists of five criteria, with scores ranging from 1 to 5. The higher the score, the better the quality [21]. The DISCERN questionnaire was used to evaluate their reliability. In addition, we used the full version of the DISCERN tool [22]. The questionnaire consists of 16 questions, scored on a 5-point scale (totaling 80 points), and is divided into three sections (the reliability of the video, the quality of treatment options, and the overall score). The total score and average value of 16 items are used to distinguish between high-quality and low-quality health information. The specific items can be found in Multimedia Appendix 1. Two independent reviewers with a background in medicine received training and independently conducted the review and evaluation. In the event of any discrepancies or disagreements between the two reviewers, a third expert was included in the discussion to determine the final score. The intraclass correlation coefficient values evaluated by the assessors were 0.901 and 0.887, indicating better reliability.

### Classification of Videos

Three groups were classified according to the source of the video, and five groups according to the content. The video sources were categorized as follows: (1) professional individuals, (2) nonprofessional individuals, and (3) professional institutions. The video content was classified into the following categories: (1) treatment description, (2) image analysis, (3) treatment suggestion, (4) precautions after intervention, and (5) experience and feeling. Specific classification criteria are shown in Multimedia Appendix 2.

### Statistical Analysis

Data analysis was performed using SPSS (IBM Corp) software. Normally distributed continuous data were expressed as $\left(\bar{\chi }\pm \left[s\right]\right)$, while skewed data were presented as median (IQR). Categorical data were presented as frequency and percentage. The normality of GQS and DISCERN scores was assessed using the Shapiro-Wilk test. For normally distributed continuous data, comparisons between groups were conducted using the independent samples 2-tailed *t* test or ANOVA. For nonnormally distributed continuous data, comparisons between groups were made using the Mann-Whitney *U* test or Kruskal-Wallis H test. Spearman correlation analysis was performed to assess the relationships between variables. Variables with *P*<.05 were selected for inclusion in the multivariate analysis. Multiple linear regression was applied to identify the factors influencing video quality. GraphPad Prism (version 9.0.2; GraphPad Software, LLC) software was used for plotting the graphs.

## Results

### Overview of the Video Screening Process

To obtain the top 100 eligible videos from Bilibili and TikTok, we removed 16 noncompliant videos from Bilibili and 25 from TikTok. In the end, a thorough evaluation was carried out on a total of 200 eligible videos, comprising content from both platforms. Each video was unique in content and uploader across the two platforms (Figure 1).

### Landscape of the TACE Videos on TikTok and Bilibili

**Figure 2.**
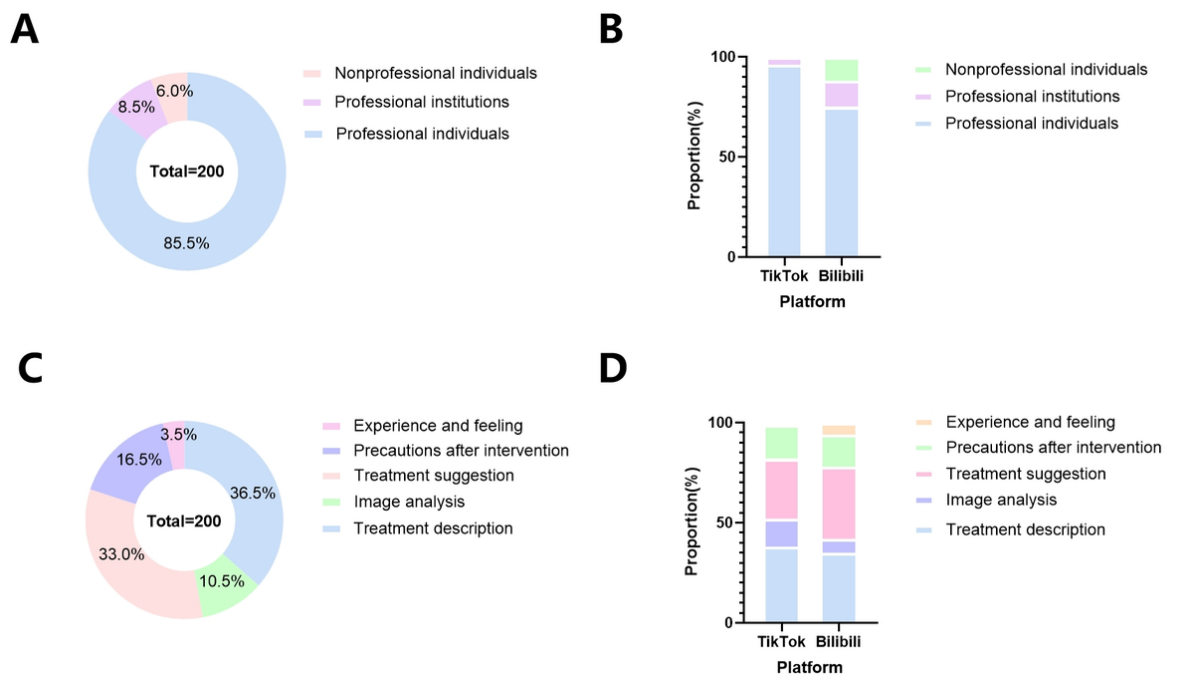
General information on TACE videos from TikTok and Bilibili. (A) The doughnut chart shows the sources of all included videos. (B) The bar chart shows the sources of videos from TikTok and Bilibili. (C) The doughnut chart shows the content of all included videos. (D) The bar chart shows the content of videos from TikTok and Bilibili. TACE: transarterial chemoembolization.

TikTok and Bilibili are public platforms in China, with a wide variety of uploaded content and types. In this study, we categorized the videos based on the uploader’s level of expertise. The results showed that videos from professionals dominated, accounting for 85.5%. The remaining sources were as follows: professional institutions (8.5%) and nonprofessional individuals (6%; Figure 2A). Overall, both TikTok and Bilibili have a large number of videos uploaded by professionals (Figure 2B). Notably, in terms of video content, “Treatment description” was the most prevalent category (36.5%), followed by “Treatment suggestions” (33%), while “Experience and feeling” had the lowest proportion (3.5%; (Figure 2C). A separate comparison of content types on TikTok and Bilibili showed similar results (Figure 2D).

### General Information and Index of Web-Based Videos

The number of views, likes, comments, shares, and other metrics on TikTok and Bilibili serve as indicators of the attention these videos receive and are suitable tools for assessing their influence. First, we compared these metrics between TikTok and Bilibili. In contrast, related videos on TikTok received more likes, comments, shares, and saves compared to those on Bilibili (*P*<.001). However, videos on Bilibili had longer durations and were uploaded earlier (*P*<.001; Table 1).

**Table 1.** Basic index of videos about TACE^a^ on Bilibili and TikTok.

Variable	TikTok (n=100), median (IQR)	Bilibili (n=100), median (IQR)	*z* ^b^	*P* value
Likes	76.00 (37.5-143)	6 (2-16)	−8.874	<.001
Comments	6 (2.25-12.75)	1 (0-3)	−6.677	<.001
Saves	17 (4.25-56.50)	4 (1-19)	−4.594	<.001
Shares	10 (2-42)	3 (1-10.75)	−3.951	<.001
Duration	1.02 (0.19-1.58)	1.54 (1.09-3.26)	−5.159	<.001
Days since published	145.50 (61-325)	582.50 (312.25-890.25)	−7.208	<.001

a TACE: transarterial chemoembolization.

b Wilcoxon rank sum test was used.

Next, we further compared the differences in the number of likes, comments, shares, saves, video duration, and upload time of web-based videos based on their source and content type (Tables2  3). In Bilibili, although nonprofessional individuals uploaded a smaller number of videos, their videos had a high median number of saves and longest durations, suggesting a potential preference among viewers for longer, personal, or experience-based content. Notably, videos from professional institutions had significantly higher median save counts and share counts, suggesting these videos may be more valued as reference material. On TikTok, the number of comments from professionals is relatively high. To our surprise, “Experience and feeling” videos showed the highest quartile value in terms of likes, though differences did not reach statistical significance. In addition, when comparing across content types, videos classified as “Treatment description” received the most saves and shares in Bilibili and TikTok. This indicates that people have a high level of attention to information related to treatment.

**Table 2.** Characteristics of the videos across sources and content in Bilibili.

Variable	Likes	Comments	Saves	Shares	Duration	Days since published
Video sources (n=100), median (IQR)						
Professional individuals (n=75)	5 (2-15)	1 (0-3)	3 (1-9)	1 (0-9)	1.46 (1.06-2.11)	505 (311-967)
Professional institutions (n=13)	8 (1.5-36)	0 (0-2)	21 (2.5-86)	13 (3-41.5)	4.38 (1.34-20.09)	584 (281.5-721)
Nonprofessional individuals (n=12)	12.5 (4-68.75)	0 (0-16)	21.5 (2-38.25)	5.5 (1.5-32.25)	12.43 (1.90-19.40)	735.5 (412.75-1039.50)
H^a^	2.135	1.303	11.863	10.827	11.774	0.365
*P* value	.34	.003	.003	.004	.003	.83
Video content (n=100), median (IQR)						
Treatment description (n=35)	11.5 (3.50-26.75)	1 (0-3)	11.5 (2.75-42.5)	9 (1.75-35.5)	2.19 (1.28-10.22)	600 (373.75-867)
Image analysis (n=7)	3 (1-6)	1 (0-2)	1 (1-2)	0 (0-1)	1.55 (0.22-3.59)	434 (141-741)
Treatment suggestion (n=36)	5 (2-13.25)	1 (0-3)	3.5 (1-13.5)	2 (1-7)	1.54 (1.14-2.06)	450.50 (130-974.5)
Precautions after intervention (n=16)	4 (2-17.25)	0 (0-3)	1 (0-6.5)	1 (0-8.75)	1.25 (0.67-4.10)	585.5 (431-813.75)
Experience and feeling (n=6)	14 (3.5-474.75)	1 (0.75-95)	2 (1-168.5)	1 (0.75-68.5)	1.29 (0.85-23.11)	584 (104.5-1245.25)
H^a^	6.908	3.368	13.933	16.210	4.164	3.611
*P* value	.14	.49	.008	.003	.38	.46

a Kruskal-Wallis H test.

**Table 3.** Characteristics of the videos across sources and content in TikTok.

Variable	Likes	Comments	Saves	Shares	Duration	Days since published
Video sources (n=100), median (IQR)						
Professional individuals (n=96)	80.5 (39.25-150.50)	6 (3-13)	18 (5-59.25)	10.5 (2-46.5)	1.02 (0.19-1.56)	145.5 (61-317.75)
Professional institutions (n=4)	25.5 (13.25-71.50)	1.5 (0.25-2.75)	9 (1.25-16.75)	3 (1-16.25)	1.54 (0.44-2.30)	305.5 (55.75-1216.75)
Nonprofessional individuals (n=0)	—^a^	—	—	—	—	—
* z* ^*b*^	−1.785	−2.257	−1.497	−0.669	−1.355	−1.566
*P* value	.07	.02	.14	.52	.19	.12
Video content (n=100), median (IQR)						
Treatment description (n=38)	98 (40.50-156.25)	6 (3-13.25)	28.25 (7.5-60.75)	22 (6-87.5)	0.83 (0.12-1.50)	279.5 (122-630)
Image analysis (n=14)	45 (17.75-68.5)	4 (1.75-7)	3 (2-7)	1.5 (1-3.5)	0.19 (0.14-0.33)	115.5 (83.5-154.75)
Treatment suggestion (n=30)	65 (36-124.25)	6.5 (2.75-13)	14.5 (6.75-53.75)	8 (3-34.5)	1.41 (0.39-3.24)	69 (26-183.25)
Precautions after intervention (n=17)	100 (30.5-535)	7 (2-42)	25 (3.5-218)	15 (1.5-131)	1.20 (0.24-2.05)	111 (67-287)
Experience and feeling (n=1)	—	—	—	—	—	—
H^c^	9.668	5.761	15.106	16.830	14.347	18.150
*P* value	.05	.22	.004	.002	.006	.001

a Views were not available on TikTok.

b Mann-Whitney *U* test.

c Kruskal-Wallis H test.

### Quality Analysis of Web-Based Videos

To objectively assess the quality of web-based videos, we conducted a thorough evaluation using the internationally recognized GQS and the DISCREN scoring system. For GQS scores, the majority of videos received a score of 2, making up 47% of the total, followed by scores of 3 (34.5%), 1 (11%), 4 (6.5%), and 5 (1%).

**Figure 3.**
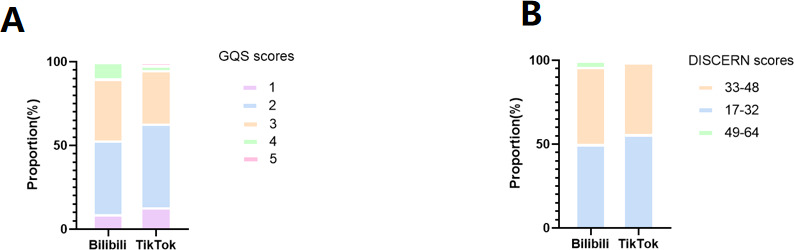
Quality analysis of web-based videos. (A) The bar chart shows the proportion of video editing software types within each GQS category (Bilibili and TikTok). (B) The bar chart shows the proportion of different software in the DISCERN scores (Bilibili and TikTok). GQS: Global Quality Score.

Regarding DISCREN scores, the range of 17‐32 represented the largest proportion at 53%, while scores of 33‐48 accounted for 44.5%, and 49‐64 for 2.5%. The quality scores of videos from both platforms were distributed primarily in the low to medium range, as shown in Figure 3 These results suggest that the quality of TACE-related videos is unsatisfactory and needs further improvement.

### Quality Comparison Across Platforms and Formats

Considering the unique characteristics of Bilibili and TikTok, we performed a comparative analysis of video quality between the two platforms. Regarding TikTok videos, the average GQS score was 2.31 (SD 0.81), and the average DISCERN score was 1.86 (SD 0.40), indicating that TikTok videos generally have moderate quality and low reliability. For Bilibili videos, the average GQS score was 2.48 (SD 0.80), and the average DISCERN score was 2.00 (SD 0.44), suggesting that Bilibili videos also have moderate quality and reliability. Furthermore, there was a significant difference in DISCERN scores between Bilibili and TikTok, with Bilibili videos being more reliable (*P*=.02). However, no significant difference was found in the GQS scores between the two platforms (*P*=.14).

To investigate whether the type of uploader affects video quality, we further compared the GQS and DISCERN scores of videos uploaded by different types of creators. The results showed no statistically significant differences in GQS scores or DISCERN ratings between different uploaders (*P*=.06; *P*=.48). However, there were differences in GQS scores and DISCERN scores between different content (*P*=.04; *P*=.04). Nevertheless, pairwise comparisons revealed no differences between the two groups (Table 4).

**Table 4.** Analysis and comparison of the quality of different sources and contents.

Variable	n	GQS^a^, mean (SD)	DISCREN, mean (SD)	*F* test (*df*)^b^	*F* test (*df*)^c^	*P* value^b^	*P* value^c^
Video sources (n=200)				2.924 (2,197)	0.730 (2,197)	.06	.48
Professional individuals	171	2.34 (0.78)	2.00 (0.48)				
Professional institutions	17	2.76 (0.75)	2.14 (0.29)				
Nonprofessional individuals	12	2.67 (1.07)	2.03 (0.38)				
Video content (n=200)				2.504 (4,195)	2.494 (4,195)	.04	.04
Treatment description	73	2.49 (0.82)	2.00 (0.44)				
Image analysis	21	2.14 (0.79)	1.74 (0.43)				
Treatment suggestion	66	2.36 (0.74)	1.94 (0.40)				
Precautions after intervention	33	2.55 (0.87)	1.96 (0.39)				
Experience and feeling	7	1.71 (0.76)	2.67 (1.07)				

a GQS: Global Quality Score.

b ANOVA test for the differences in GQS among different projects.

c ANOVA test for the differences in DISCREN among different projects.

### Correlation Analysis

Spearman correlation analysis was conducted to explore the relationships between various video variables. Positive correlations were found among the following variables: likes and comments (*r*=0.864; *P*<.001), likes and shares (*r*=0.631; *P*<.001), likes and saves (*r*=0.921; *P*<.001), saves and comments (*r*=0.734; *P*<.001), comments and shares (*r*=0.594; *P*<.001), and saves and shares (*r*=0.750; *P*<.001). Additionally, saves, shares, duration, and days since publication were positively correlated with both GQS and DISCERN. Furthermore, a strong correlation between GQS and DISCERN was observed, confirming their significant consistency and reinforcing the robustness of our scoring results (Table 5).

**Table 5.** Spearman correlation analysis between the video variables and GQS^a^ scores and DISCERN scores.

Variable	Likes	Comments	Saves	Shares	Duration	Days since published	GQS	DISCERN scores
Likes	1	0.864^b^	0.921^b^	0.631^b^	−0.015	−0.024	0.148	0.137
Comments	0.864^b^	1	0.734^b^	0.594^b^	0.032	0.074	0.130	0.120
Saves	0.921^b^	0.734^b^	1	0.750^b^	0.010	0.030	0.189^b^	0.222^c^
Shares	0.631^b^	0.594^b^	0.750^b^	1	−0.018	0.076	0.194^b^	0.209^b^
Duration	−0.015	0.032	0.010	−0.018	1	0.141	0.164^c^	0.267^b^
Days since published	−0.024	0.074	0.030	0.076	0.141	1	0.216^b^	0.238^b^
GQS scores	0.148	0.130	0.189^b^	0.194^b^	0.164^c^	0.216^b^	1	0.802^b^
DISCERN scores	0.137	0.120	0.222^c^	0.209^b^	0.267^b^	0.238^b^	0.802^b^	1

a GQS: Global Quality Score.

b Significant at *P*<.001

c Significant at *P*<.05

### An Investigation Into Determinants of Video Quality

By incorporating meaningful factors from univariate and correlation analyses into multivariate analysis, the results showed that the number of saves and the number of days since upload were closely related to GQS. Specifically, videos with a higher number of saves and longer time since upload tended to have higher GQS quality. Additionally, the number of saves, video duration, and number of days since upload were closely related to DISCERN. Specifically, videos with more saves, longer duration, and earlier upload times demonstrated better reliability (Table 6).

**Table 6.** Multiple linear regression analysis of GQS^a^ scores and DISCERN scores.

Variable	B	SE	β	*t* test (df)	*P* value
GQS					
Constants	2.149	0.084	—^b^	26.304 (197)	<.001
Days since published	<.001	<0.001	0.214	3.127 (197)	.002
Saves	.001	<0.001	0.184	2.685 (197)	.008
DISCERN					
Constants	1.804	0.043	—	41.487 (196)	<.001
Duration	.005	0.001	0.213	3.133 (196)	.002
Days since published	<.001	<0.001	0.168	2.50 (196)	.01
Saves	<.001	<0.001	0.176	2.595 (196)	.01

a GQS: Global Quality Score.

b Not applicable.

## Discussion

### Overview

TACE treatment usually requires multiple surgeries for patients. Therefore, the patient’s knowledge, attitude, and treatment compliance are crucial for the success of the treatment. In this regard, educational videos on social media provide a convenient means of accessing relevant information, helping to overcome the traditional barriers to obtaining medical support and resources. Several studies, including those on attention deficit [23], laryngeal cancer [24], thyroid cancer [25], and gallstones [20], provide evidence that short videos facilitate easy access to and positive dissemination of disease-related information. However, the overall quality of these videos is unsatisfactory, raising concerns about misinformation on social media. Inaccurate or misleading information spreads rapidly on these platforms, potentially having a negative impact on health-related attitudes and behaviors. To evaluate the quality and identify the factors influencing TACE-related videos, we performed a comprehensive content analysis of these videos.

### Principal Findings

This study is the first to comprehensively evaluate TACE-related videos across major video platforms. We reviewed the top 100 short videos on TACE from two popular Chinese platforms, TikTok and Bilibili. In this cross-sectional study, the general characteristics of these videos were analyzed, the DISCERN and GQS tools were used to assess their quality and reliability, and the factors influencing video quality were investigated. Our findings reveal that the overall quality of short videos on both platforms was lower than anticipated. Furthermore, we identified that factors such as the number of saves and the number of days since upload were associated with video quality. Additionally, the number of saves, video duration, and days since upload were linked to video reliability.

### Video Characteristics

We found that TikTok is more popular than Bilibili. To be precise, the number of likes, comments, shares, and favorites is all high. However, videos on Bilibili tend to have longer durations and earlier upload times. Research indicated that shorter videos were generally more engaging and spread more quickly, which helped explain why TikTok videos received more likes despite having shorter durations [26]. Additionally, we analyzed the types of video creators and found that most TACE-related videos on both platforms were uploaded by professionals. On the other hand, we analyzed the different types of TACE-related content on both platforms. Treatment descriptions were the most prevalent. This underscores the strong demand for treatment-related knowledge among patients with HCC. We observed “experience and feeling” elicited higher levels of interaction, including shares and saves, compared to more technical formats like “image analysis.” This implies that emotionally engaging or explanatory content may be more effective in attracting user attention and encouraging dissemination. However, videos describing treatment experiences and personal feelings were the least prevalent. This highlights a gap in TACE-related content on TikTok and Bilibili. Therefore, we encourage health care professionals to expand and diversify video content to include aspects such as prevention, treatment, and precautions, ensuring that the public receives comprehensive and reliable medical information.

### Quality of the Short Videos on TACE

The quality and reliability of TACE-related short videos on both platforms were found to be low, with overall performance primarily falling within the moderate to poor range. Research has shown that inaccurate and misleading videos on social media often receive more views from users compared to accurate content, which is similar to our research findings [27,28]. While watching the videos, we noticed that in order to draw attention, some nonprofessionals pretended to be professionals and promoted unproven and unreliable treatment methods. What surprised us was that these videos received a high number of likes and saves. Due to the complexity of the surgery and the psychological burden associated with liver cancer treatment, inaccurate or misleading video content regarding TACE may have serious consequences [29,30]. The patient may thus develop unrealistic expectations regarding such matters, underestimate potential complications, or improperly delay seeking clinical advice. Furthermore, incorrect information may erode people’s trust in health care professionals, especially when professional sources are perceived as less appealing than nonprofessional ones [30]. This highlights the urgent need for a balanced health communication strategy that combines accuracy, clarity, and engagement.

Improving health content quality requires a multilevel strategy. First, the platform can modify its algorithm to enhance the visibility of content provided by verified medical professionals and certification organizations, especially in searches related to disease terms. Second, social media companies can establish professional health content review teams, and these teams should possess basic medical knowledge and the ability to judge health literacy. Third, the platforms lack scientific oversight from experts in relevant fields and are unable to conduct peer reviews of the videos being published and shared. It is regrettable that implementing peer reviews may not be feasible. They can assist these platforms in developing effective tagging systems to flag potentially inaccurate or harmful health content. Finally, it is recommended that health care professionals provide evidence-based information when posting videos, including links or citations to credible sources.

We further analyzed the factors related to the quality and reliability of the video content. Interestingly, we found that video duration was closely related to content reliability. This result is likely related to platform limitations. Short video platforms have time constraints, making it difficult to provide the necessary background or depth to convey a complete picture or concept within such a short period. The lack of sufficient information somewhat reduces the quality of the content. Moreover, viewers are often unable to distinguish between high-quality and low-quality videos [13,20]. The audience for these short videos is not composed of professional health care workers, and they often find specialized knowledge unengaging, which leads to content being simpler and more superficial. Additionally, although there were no significant differences in video quality between the two platforms, there were notable differences in reliability, with Bilibili’s content being rated as more reliable. This result may also be related to video duration. Bilibili allows for more flexibility in video length, enabling clearer presentation of treatment-related information, while TikTok typically has shorter video durations. Therefore, given the complexity and specialization of medical content, the length of medical-related videos should be extended accordingly to ensure that sufficient information is provided. Furthermore, health care professionals should seek diverse forms of video content to increase its richness and engagement. Interestingly, we found that the longer the video has been published, the better the content quality and reliability. This serves as a warning: the quality of content tended to decrease. Given the widespread focus on entertainment and viral trends, the importance of video content should not be overlooked.

### Strengths and Limitations

This study has several strengths. First, it is the first study to analyze the quality of short videos related to TACE on social media platforms, offering guidance for the public in accessing health information and providing valuable insights for content creators and platforms. Second, our research focused on China’s two largest short video platforms, overcoming the limitations of studying a single platform, which enhances the reliability of our results. Finally, we used widely recognized tools, the GQS and DISCERN, to assess the quality and reliability of the videos, ensuring a robust evaluation of our findings.

However, this study has some limitations. First, the videos included in our analysis were limited to those from China, and global platforms like YouTube, which dominate the web-based video space, were excluded due to access restrictions. Second, we focused on the top 100 videos from each platform. This algorithm does not equate to relevance or quality. It may systematically overlook content with lower visibility but potentially higher accuracy. Future research should consider broader sampling strategies to capture less visible but potentially high-quality content. Third, due to the limited sample size within certain categories, we did not perform subgroup or interaction analyses, which might have missed some subtle differences. We identified it as a priority for future, larger-scale analyses.

### Conclusions

The overall quality and reliability of the top 100 videos related to TACE videos on TikTok and Bilibili were relatively low. Higher numbers of saves and longer time since upload were positively associated with better video quality and reliability. These findings highlight the need for caution when using social media as a health information source. The quality of the video should be further improved. Platforms should adjust their algorithms to prioritize content from verified medical professionals. Establishing review teams with basic medical literacy can help flag misleading information. Health care professionals are also encouraged to share evidence-based information and cite credible sources when publishing videos.

## Supplementary material

10.2196/73855 Multimedia Appendix 1 Full list of questions.

10.2196/73855 Multimedia Appendix 2 Classification of videos.

## Abbreviations

GQS: Global Quality Score
HCC: hepatocellular carcinoma
TACE: transarterial chemoembolization
