# Supplementary material for: Quality and Reliability of Transarterial Chemoembolization Videos on TikTok and Bilibili: Cross-Sectional Content Analysis Study
Source: JMIR Form Res. 2025 Sep 17;9:e73855. doi: 10.2196/73855 (PMC12443354; doi:10.2196/73855)
Supplement: Multimedia Appendix 1 [file formative-v9-e73855-s001.docx]

| 1. Are the aims clear? |
| --- |
| 2. Does it achieve its aims? |
| 3. Is it relevant? |
| 4. Is it clear what sources of publications were used to compile the publication (other than the author)? |
| 5. Is it clear when the information used or reported in the video was produced? |
| 6. Is it balanced and unbiased? |
| 7. Does it provide details of additional sources of support and information? |
| 8. Does it refer to areas of uncertainty? |
| 9. Does it describe how each treatment works? |
| 10. Does it describe the benefits of each treatment? |
| 11. Does it describe the risks of each treatment? |
| 12. Does it describe what would happen if no treatment is used? |
| 13. Does it describe how the treatment options affect quality of life? |
| 14. Is it clear that there may be more than one possible treatment choice? |
| 15. Does it provide support for shared decision-making? |
| 16. Based on the answers to all of the above questions, rate the overall quality of the publication as a source of information about treatment choices |

Full list of questions in the DISCERN Instrument

Table S3: Full list of questions in the GQS Instrument

| Scale | Description |
| --- | --- |
| 1 | Poor quality, poor flow of the site, most information missing, not at all useful for patients |
| 2 | Generally poor quality and poor flow, some information listed but many important topics missing, of very limited use to patients |
| 3 | Moderate quality, suboptimal flow, some important information is adequately discussed but others poorly discussed, somewhat useful for patients |
| 4 | Good quality and generally good flow, most of the relevant information is listed, but some topics not covered, useful for patients |
| 5 | Excellent quality and excellent flow, very useful for patients |
