# Supplementary material for: Quality and Reliability of Transarterial Chemoembolization Videos on TikTok and Bilibili: Cross-Sectional Content Analysis Study
Source: JMIR Form Res. 2025 Sep 17;9:e73855. doi: 10.2196/73855 (PMC12443354; doi:10.2196/73855)
Supplement: Multimedia Appendix 2 [file formative-v9-e73855-s002.docx]

|  | |
| --- | --- |
| Classification | Definition |
| Video Source | |
| 1. Professional Individuals | Licensed healthcare professionals (e.g., physicians, nurses) who shared videos using personal accounts and whose professional credentials (e.g., titles, affiliations, medical certifications) were verifiable through their platform profiles or external sources |
| 1. None-professional Individuals | People who lack real name recognition or professional credentials in the medical field |
| 1. Professional Institutions | Videos uploaded through the official accounts of hospitals, medical universities or certified health organizations usually bear the institution's logo, the certified emblem or the organization's description |
| Video Content | |
| 1. Treatment description | Videos that provide factual and general information about TACE, including but not limited to its pathological basis, mechanism of action, clinical indications, procedural steps, advantages, disadvantages, and success rates. These videos do not contain personalized advice or specific recommendations |
| 1. Image analysis | Focused on interpreting imaging results related to TACE, such as CT and enhanced CT were analyzed |
| 1. Treatment suggestion | Videos that offer subjective opinions, advice, or guidance on whether patients should choose TACE, what specific treatment plan to follow, or comparisons with alternative treatments. These may include patient-specific recommendations, clinician opinions, or lay advice intended to influence decision-making. |
| 1. Precautions after intervention | Precautions after TACE, including the prevention and treatment of complications and adverse reactions, postoperative review, diet, drugs, etc |
| 1. Experience and feeling | Subjective narratives describing patients’ feelings or recovery process |
